# Advanced chronic kidney disease coexisting with heart failure: navigating patients’ management

**DOI:** 10.1093/ckj/sfaf128

**Published:** 2025-05-02

**Authors:** Carmine Zoccali, Adeera Levin, Francesca Mallamaci, Robert Giugliano, Raffaele De Caterina

**Affiliations:** Renal Research Institute, New York, NY, USA; Institute of Molecular Biology and Genetics (Biogem), Ariano Irpino, Italy; Associazione Ipertensione Nefrologia Trapianto Renal (IPNET), c/o Nefrologia, Grande Ospedale Metropolitano, Reggio Calabria, Italy; Division of Nephrology, Department of Medicine, University of British Columbia, Vancouver, British Columbia, Canada; Associazione Ipertensione Nefrologia Trapianto Renal (IPNET), c/o Nefrologia, Grande Ospedale Metropolitano, Reggio Calabria, Italy; Cardiovascular Division, Brigham and Women's Hospital, Harvard Medical School, Boston, USA; Chair of Cardiology, University of Pisa and Cardiology Division, Pisa University Hospital, Pisa, Italy

**Keywords:** care models, chronic kidney disease, heart failure, therapies

## Abstract

Chronic kidney disease (CKD) and heart failure (HF) are interrelated, mutually exacerbating conditions. HF in patients with moderate to severe CKD poses unique clinical problems. Indeed, considerations related to specific concomitant derangements, such as vascular calcification, inflammation, and proteinuria, inform and demand personalized treatment strategies. Pharmacological interventions, including renin–angiotensin system antagonists, sodium-glucose cotransporter 2 (SGLT2) inhibitors and novel mineralocorticoid receptor blockers are valuable in managing these complex conditions, although frequently difficult or impossible to use in advanced kidney disease. Precision medicine, innovative treatments, and the incorporation of digital health tools, artificial intelligence, remote monitoring, and advanced imaging techniques into patient care are redesigning the scenario of HF associated with CKD. AI-driven predictive analytics for early detection of decompensation, telemedicine for remote consultations, and electronic health records with decision-support systems. These innovations enhance personalized treatment, improve early intervention, and optimize disease management, ultimately leading to better outcomes for patients with HF and CKD. Collaborative care models are being implemented and evaluated to advance the management of such conditions. Thus, the integration of novel therapeutic approaches and personalized medicine holds promise for improving patient outcomes, while ongoing research is essential to enabling innovation in this area. Here we review the current management of concomitant kidney disease and HF, highlighting areas for proposed future refinements.

## INTRODUCTION

Chronic kidney disease (CKD) and heart failure (HF) are interdependent conditions that significantly impact patient outcomes, each exacerbating the progression of the other. This relationship necessitates a nuanced approach to management, particularly in advanced CKD stages (G3b or higher), where the intensified complexity of care demands a particularly careful approach. The pivotal role of estimated glomerular filtration rate (eGFR) and urine albumin to creatinine ratio (uACR) in risk stratification in HF underscores the importance of kidney function and damage in clinical decision-making for patients with HF. However, the chronic management of HF in the context of CKD remains fraught with challenges.

Numerous studies and a systematic review have addressed the acute setting of HF in CKD patients [1]. Less well described are specific issues related to chronic management of HF in patients with advanced CKD, which requires a long-term, multifaceted approach involving symptom management, prevention of disease progression on both sides, as well as minimization of the damage that each condition imposes on the other.

Here, after briefly touching on epidemiology, prognosis, and classification issues, we discuss the recent literature on a series of problems specifically challenging the management of patients with HF and CKD, delving into several pressing issues. These include the management of electrolyte imbalances exacerbated by therapies such as renin–angiotensin–aldosterone (RAAS) inhibitors and diuretics, and the difficulties in assessing and optimizing volume status. We here discuss strategies to address these imbalances, emphasizing the need for careful monitoring and individualized treatment adjustments. Furthermore, we examine the integration of novel therapeutic approaches, such as SGLT2 inhibitors and mineralocorticoid receptor antagonists, offering promising benefits in this patient population. We also consider here the role of innovative technologies, including telemedicine and remote monitoring, in enhancing personalized care and early intervention. We emphasize that a multidisciplinary approach and the exploitation of emerging therapies and technologies may lead to improved outcomes for both the heart and kidney.

## EPIDEMIOLOGY, PROGNOSIS, CLASSIFICATION

The Global Burden of Disease Study underlines that ischemic heart disease, a primary cause of HF, and CKD are both age-dependent, and estimates that they rank as the first and the sixteenth leading causes of death worldwide [2]. Of patients hospitalized for HF, more than half have moderate (43%) or severe (13%) CKD [3]. HF is the first cause of hospitalization among older adults and contributes significantly to healthcare costs [4]. Both CKD and HF reduce life expectancy: patients with advanced CKD have a higher all-cause and cardiovascular mortality compared to those with normal kidney function [5]. Similarly, HF is linked to high morbidity and mortality, with a 5-year survival rate of ∼50% after diagnosis. Patients with both conditions have at least an additive risk of hospitalization and death compared to those with either condition alone [6]. The presence of CKD in HF patients is associated with a 2- to 3-fold increase in mortality, underscoring the need for improved understanding of appropriate and timely initiation of evidence-informed management strategies. In addition, these conditions require frequent hospitalization, complex management, and long-term care, leading to substantial healthcare costs.

Table 1 shows the current classifications of CKD and HF according to the 2024 Kidney Disease: Improving Global Outcomes organization (KDIGO) guidelines and the 2022 ACC/AHA and 2021 ESC guidelines.

**Table 1:** Current classifications of CKD and HF.

Aspect	CKD	HF
Classification system	KDIGO 2024 Guidelines	AHA, ACC, HFSA, ESC Guidelines
Dimensions	Cause, eGFR, albuminuria	Ejection fraction, physical symptoms
Stages	Stage G3b: moderate to severe reduction in eGFR (30–44 ml/min/1.73 m²)	• Stage A: risk factors without structural heart disease • Stage B: structural heart disease, no symptoms • Stage C: symptoms present • Stage D: advanced disease
Additional risk prediction tools	• Kidney Failure Risk Equation • SCORE2 • SCORE2-OP	Ejection fraction categories: HFrEF, HFpEF, HFmrEF
Global impact	12th leading cause of death worldwide	Affects >64 million people globally
Prevalence in hospitalized patients	43% moderate, 13% severe CKD in HF patients	Leading cause of hospitalization among older adults
Mortality and morbidity	Higher all-cause and CV mortality in advanced CKD	5-year survival rate of ∼50% after diagnosis
Combined impact	Increased risk of hospitalization and death when both CKD and HF are present	2–3-fold increase in mortality with CKD
Healthcare costs	Substantial due to frequent hospitalizations and long-term care	Significant contribution to healthcare costs

## CHALLENGING TREATMENT PROBLEMS OF HEART FAILURE IN CHRONIC KIDNEY DISEASE

**Figure 1:**
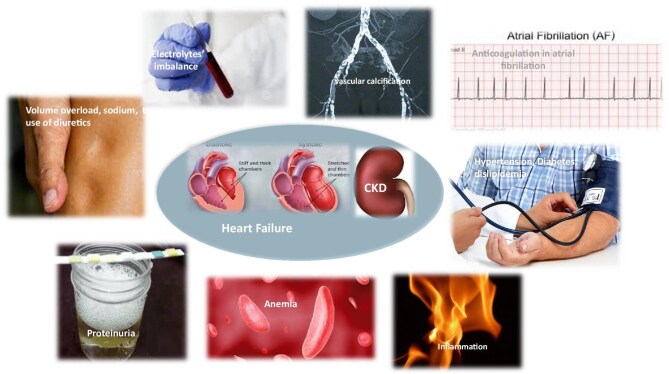
Particular issues impact HF patients with CKD. The main text discusses these issues clockwise, from volume overload, sodium, electrolyte imbalances, and the use of diuretics.

Patients with HF and CKD face many complex and interrelated problems that require an in-depth assessment of etiology, severity, and comorbidities to develop the most appropriate therapeutic program (Fig. 1, Tables 2**–**5).

**Table 2:** Dosages of drugs used for HF in patients with coexisting renal disease.

Drug	eGFR >60 ml/min/1.73 m^2^	eGFR 30–60 ml/min/1.73 m^2^	eGFR <30 ml/min/1.73 m^2^	Warnings
ARNI Sacubitril-valsartan	49 mg/51 mg to 97 mg/103 mg, twice a day.		Initial: Sacubitril 24 mg/valsartan 26 mg twice daily. Double the dose as tolerated every 1 to 2 weeks to the target maintenance dose of sacubitril 97 mg/valsartan 103 mg twice daily.	Hypotension, hyperkaliemia, eGFR reduction. Safety and efficacy data are limited in this population, especially at eGFR <20 ml/min/1.73 m^2^.
			The same scheme can be used in dialysis patients.	
Beta-blockers				
Bisoprolol	Single dose of 2.5 mg to 10 mg/day.		No dose adjustment	
Metoprolol	12.5–25 mg extended release once daily; up-titrate gradually to a target dose of 200 mg once daily.			
Carvedilol	Initial: 3.125 mg twice daily; up-titrate gradually to 25 mg twice daily (≤85 kg) or 50 mg twice daily (>85 kg).		Poorly dialyzable, 3.25 mg with no up titration.	Try before ACEi. Monitor heart rhythm.
ACE inhibitors				Hypotension, hyperkaliemia, eGFR reduction.
Captopril	Initial: 6.25 mg 3 times daily; increase dose (e.g. double) as tolerated to a target dose of 50 mg 3 times daily.	Creatinine clearance (Cr Cl) ≥50 ml/min: no dosage adjustment.	CrCl <10 initial: 6.25 mg every 24 h. Titrate slowly to a maximum dose of 50 mg every 24 h.	
		CrCl 10 to <50 ml/min: initial: 6.25 mg every 12 to 24 hours. Titrate slowly to a maximum dose of 50 mg every 12 hours.		
Lisinopril	Initial: 2.5 to 5 mg once daily; increase dose as tolerated to a target dose of 20 to 40 mg once daily.	CrCl ≥30 ml/min: no dosage adjustment.	CrCl <10 ml/min: consider alternative therapy.	
			Hemodialysis initial: 10 mg 3 times weekly post hemodialysis; titrate to a maximum of 40 mg 3 times weekly. In PD patients: standard dosing.	
Ramipril	Single dose of 2.5 mg gradually uptitrated to a maximum dose of 10 mg/day.	No dose adjustment.	eGFR 15 to <30 ml/min/1.73 m^2^: initial: 1.25 mg once daily; up-titrate gradually to a maximum dose: 5 mg/day in 1 or 2 divided doses.	
			eGFR <15 ml/min/1.73 m^2^. Use of alternative agents should be considered.	
			Can be used in dialysis patients.	
Enalapril	Initial: 2.5 mg twice daily; increase dose (e.g. double) as tolerated every ≥1 to 2 weeks to a target dose of 10 to 20 mg twice daily.	CrCl ≥30 ml/min: no dosage adjustment.	CrCl 10 to 30 ml/min: initial: 2.5 mg daily in 1 or 2 doses; titrate slowly to a maximum recommended dose: 20 mg/day.	
			CrCl <10 ml/min: consider alternative therapy.	
Quinalapril	Initial: 5 mg twice daily; up titrated to a target dose of 20 mg twice daily.	CrCl ≥30 ml/min: initial dose 5 mg once a day. CrCl 10–30 ml/min: 2.5 mg once a day.	Hemodialysis initial: 2.5 mg 3 times weekly administered post dialysis; titrate slowly to a maximum recommended dose: 10 mg once daily.	
			CrCl <10 ml/min: no dosage adjustment provided in the manufacturer's labeling.	
Angiotensin II receptor blockers (ARBs)				Hypotension, hyperkaliemia, eGFR reduction.
Losartan	Initial: 25 to 50 mg once daily; increase gradually to a target dose of 150 mg once daily.	No dose adjustment.	No dose adjustment is necessary in advanced CKD and in dialysis patients.	
Valsartan	40 to 160 mg twice a day.	No dose adjustment.	There are no dosage adjustments provided in the manufacturer's labeling; has not been studied; valsartan use in CKD undefined.	
Irbesartan	150 mg once daily; if needed up-titrate to 300 mg once daily.	No dose adjustment.	No dose adjustment is necessary in advanced CKD and in dialysis patients.	
Candesartan	4 to 8 mg once daily; gradually increase dose up to a target dose of 32 mg once daily.	CrCl ≥30 ml/min: No dosage adjustment.	CrCl <30 ml/min and hemodialysis and PD patients: initial dose 4 mg once a day. Titrate gradually based on tolerability.	
Mineralo corticoid receptor antagonists (MRS)				
Eplerenone	Single dose of 25 to 50 mg/day.	eGFR 31 to 49 ml/min/1.73 m^2^: Initial: 25 mg every other day; if tolerated, dose may be increased to 25 mg once daily.	Contraindicated when the eGFR is < 30 ml/min/1.73 m^2^.	Hypotension, hyperkaliemia,
Finerenone	20 mg once daily.	eGFR ≥25 to <60 ml/minute/1.73 m^2^: 10 mg once daily.	Contraindicated when eGFR <25 ml/min/1.73 m^2^.	
SGLT2i				
Empaglifozin	Single dose of 10 mg/day.	Can be used when eGFR ≥20 ml/min/1.73 m^2^.	Still no information on the use for HF treatment in dialysis patients.	Hypoglycemia, excessive volume depletion, Fournier's gangrene, and ketoacidosis.
Dapaglifozin	Single dose of 10 mg/day.	Can be used when eGFR ≥25 ml/min/1.73 m^2^.		
Caniglifozin	100 mg once daily.	Can be used when eGFR ≥30 ml/min/1.73 m^2^.		
Ertuglifozin	5 mg once daily.	Can be used when eGFR ≥45 ml/min/1.73 m^2^.		
Vericiguat (may be considered for additional therapy in patients who are persistently symptomatic despite an optimal medical regimen for HFrEF)	Initial: 2.5 mg once daily with food; double the dose every ≥2 weeks to a target maintenance dose of 10 mg once daily.	eGFR ≥15 ml/min/1.73 m^2^ no dose adjustment.	eGFR <15 ml/min/1.73 m^2^No dosage adjustments provided in the manufacturer's labeling.Hemodialysis and PD patients: no dose adjustment when used short term.	Low BP, anemia.
				
Hydralazine/nitrates (might be considered to reduce morbidity and mortality in patients with current or previous symptomatic HFrEF who cannot be given first-line agents, i.e. ARNI, ACEi, ARBS, SGLT2i and MRAs)	Hydralazine initial: 25 mg 3 times daily in combination with isosorbide dinitrate; titrate dose as tolerated to a target dose: 75 or 100 mg 3 times daily.	eGFR ≥10 ml/min/1.73 m^2^ no dose adjustment when used short term.	Hemodialysis and PD patients: no dose adjustment.	Headache, loss of appetite, nausea, vomiting, diarrhea, fast heart rate, and chest pain.
	Isosorbide dinitrate initial: 20 mg 3 times daily in combination with hydralazine; titrate dose as tolerated up to 40 mg 3 times daily.	No dosage adjustment necessary for any degree of kidney dysfunction.		Headaches, dizziness or light-headedness, flushing or a warm feeling in the face.
Ivabradine (may be considered for additional therapy in patients who are persistently symptomatic despite an optimal medical regimen for HFrEF)	Heart rate >60 bpm: Increase dose by 2.5 mg twice daily; maximum dose: 7.5 mg twice daily.	CrCl ≥15 ml/min: No dose adjustment necessary.	CrCl <15 ml/min: oral: initial: 2.5 mg twice daily; may increase dose based on tolerability.	Irregular or slow heartbeat, low BP.
	50 to 60 bpm: Maintain dose.<50 bpm or signs and symptoms of bradycardia: Decrease dose by 2.5 mg twice daily; if current dose is 2.5 mg twice daily, discontinue therapy.			

Dosages of drugs listed in this table were taken from UpToDate (https://www.wolterskluwer.com/en/solutions/uptodate). Information on kidney function is reported as estimated GFR (eGFR) or Creatinine Clearance (Cr.Cl) depending on the information in the source studies.

**Table 3:** Problems related to management of volume and electrolyte imbalance and mineral metabolism in HF patients with CKD.

Topic	Key points	Management strategies	Comments	References
Volume status management				
Clinical monitoring	Daily weight tracking, physical exams, BIS analysis, lung ultrasound	Multidisciplinary approach	Involves cardiologists and nephrologists to optimize treatment	
BIS analysis	Identifies fluid overload and depletion, limited by variability and patient factors	Used to monitor fluid status	Not recommended for single measurements in individual patients	[13]
Lung ultrasound	Guides HF treatment, reduces hospitalization risk, requires conditional interpretation	Safe and effective method	Useful in guiding treatment, especially in severe HF	[14]
Sodium restriction				
	Strict sodium restriction not beneficial in HF	A less stringent sodium target (3 g/day) seems rationale but there is no clinical trial testing this possibility	The risk for CV events in CKD patients flattens out at about 3 g sodium excretion/day	[19]
Use of diuretics and MRAs				
	Diuretics crucial in HF management	Loop diuretics preferred in CKD stage 4	Combining diuretics can be effective but increases risk of imbalances	[7, 70, 31, 71]
Electrolyte imbalance				
Hyperkalemia	Reduced renal excretion, use of RAAS inhibitors and mineralocorticoid receptor antagonists	Dietary potassium restriction, potassium binders	Requires careful monitoring to prevent cardiac arrhythmias	[72, 73]
Hypokalemia	Use of diuretics	Potassium supplementation, adjustments in diuretic therapy	Important to prevent arrhythmias and worsening HF symptoms	
Hyponatremia	Fluid overload, use of diuretics	Fluid restriction, adjustment of diuretic therapy	Critical to reduce morbidity and mortality	[34–37]
Abnormalities of mineral metabolism, vascular disease				
Hypercalcemia	Prevalence 4% in HF patients (Danish Registries)	Loop diuretics	Prevent vascular calcification initiation and progression and short- and long-term risk death risk	[39]
Hypocalcemia	Prevalence 33% in HF patients (Danish Registries)	Calcium-based phosphate binders, vitamin D analogs	Prevent short- and long-term risk death risk	[39]
Hyperphosphatemia	Associated with LVH, which may worsen HF	Phosphate binders	Akey factor in secondary hyperparathyroidism	[40]
Hypomagnesemia	Use of diuretics	Magnesium supplementation	Necessary to prevent arrhythmias and sudden death	[48]
Vascular calcification	A main factor in arterial stiffness in CKD. Predicts survival and cardiovascular events. CAS prevalence in CKD is 27% or higher	Drugs used to manage hyperphosphatemia and secondary hyperparathyroidism	No randomized trial aimed at modifying vascular calcification has shown favorable effects on major clinical outcomes in CKD	[43, 45]

**Table 4:** Problems related to anticoagulation in AF and to the treatment of hypertension, diabetes, and hyperlipidemia.

Topic	Key points	Management strategies	Comments	References
Anticoagulation in atrial fibrillation	AF increases thromboembolic risk in HF and CKD. Warfarin requires monitoring; DOACs offer fixed dosing.	Apixaban can be used down to an eGFR >15 ml/min/1.73 m^2^. Dosage reduced if at least 2/3 criteria: creatinine <1.5 mg/dl, age >80 years, weight <60 kg. Rivaroxaban (at a reduced dosage when the eGFR is <30 ml/min/1.73 m^2^) and Edoxaban (at a reduced dosage when 1/3 of the following: eGFR < 50 ml/min/1.73 m², weight < 60 kg, or concomitant use of other drugs) can be used down to a eGFR level >15 ml/min/1.73 m^2^. Dabitragan can be used down to a an eGFR >30 ml/min/1.73 m^2^.	Three DOACs trials in CKD were small and inconclusive. Balance stroke risk against bleeding risk, considering patient factors.	[49–51]
Hypertension	Hypertension is common in HF and CKD, challenging to determine optimal BP targets, especially with high arterial stiffness and vascular calcification.	BP target of <130/80 mmHg suggested for HF and ASCVD prevention. Drugs with outcome benefits and BP-lowering effects recommended.	Intensive BP control may not benefit patients with moderate-to-advanced CKD.	[7]
Diabetes and Dyslipidemia	Diabetes complicates HF and CKD management. Glycemic control is crucial.	SGLT2i and GLP-1 RA recommended based on kidney function. Statins are first-line treatments recommended for cardiovascular risk reduction.	Tailor therapy to kidney function to maximize benefits and minimize risks.	[57, 59]

**Table 5:** Problems related to inflammation, anemia, and proteinuria.

Topic	Key points	Management strategies	Comments	References
Increased Proteinuria	Proteinuria is a marker of kidney damage and cardiovascular risk.	RAAS inhibitors and SGLT2 inhibitors reduce proteinuria and HF risk.	Management must balance benefits with risks of hyperkalemia and renal function worsening.	[74–76]
Anemia	Anemia is prevalent in HF and CKD. ESAs improve anemia but may not provide significant clinical benefits.	Iron supplementation is critical. Intravenous iron reduces HF hospitalizations and cardiovascular mortality.	Guidelines recommend iron studies and intravenous iron replacement for iron deficiency in HF.	[7]
Inflammation	Chronic inflammation is a hallmark of HF and CKD and a forerunner of vascular calcification.	The pillars of HF treatment impact inflammation. Novel agents like IL-1β antagonist canakinumab still untested in HF.	Important to counter inflammation to prevent vascular calcification.	[63]

The primary therapy component in patients with HF with reduced ejection fraction (HFrEF) with New York Heart Association class II to III HF symptoms is a combination therapy with one agent from each of the four classes of medications. These include beta-blockers, RAAS antagonists, mineralocorticoid receptor antagonists (MRA), and sodium-glucose cotransporter 2 inhibitors (SGLT2i). The angiotensin receptor-neprilysin inhibitor (ARNI, i.e. sacubitril-valsartan) is now first-line recommendation in HFrEF by both the American Heart Association (AHA)/American College of Cardiology (ACC) [7] and the European Society of Cardiology [8] guidelines. This drug combination has also been tested in hemodialysis patients, with a favorable impact on all-cause mortality and hospitalization [9]. A scheme of dose adjustments and warnings of commonly prescribed drugs of these classes is presented in Table 2.

Early declines in eGFR are common after initiating renin–angiotensin system inhibitors [10], ARNIs [11], and SGLT-2 inhibitors [12], often reflecting hemodynamic changes rather than true kidney injury. These declines—typically up to 20%–30% for RAS inhibitors and ARNIs, and by only 3–6 ml/min/1.73 m² for SGLT-2 inhibitors—are generally acceptable and should not prompt premature discontinuation. Clinicians should start at low doses (except for SGLT-2 inhibitors, which require no titration), up-titrate them gradually, and monitor renal function, potassium, and blood pressure at baseline, within 1–4 weeks of initiation or dose changes, and every 3–6 months thereafter. Recognizing these expected changes and monitoring them appropriately helps prevent adverse events while preserving their long-term cardiorenal benefits.

### Challenges in managing volume status (Table 3)

The assessment and monitoring of volume status are central to preventing complications in CKD, particularly in the presence of HF. To this end, patients are instructed to track their weight daily for an early detection of fluid retention. Signs of fluid overload, such as changes in lung auscultation, jugular venous distension, and peripheral edema, are first-line clinical manifestations of volume overload. However, crackles and rales at lung auscultation are poorly associated with objective measurements of fluid overload in the lung, the most critical area of the central circulation. An accurate assessment of volume status using clinical tools in HF patients with CKD is problematic, as is the interpretation of biochemical markers traditionally used, such as brain natriuretic peptide (BNP) and N terminus pro-BNP. Various technologies, including bioimpedance spectroscopy (BIS) [13] and, particularly, cardiac ultrasound—a technique that can be applied at the point of care—are promising but not yet well incorporated into clinical practice. There is some evidence that a better assessment of volume overload is possible with lung ultrasound (LUS). In 2023, a clinical Consensus Statement from the European Association of Cardiovascular Imaging on LUS in acute and chronic HF [14] on applying LUS in various conditions, including HF, has promoted this point-of-care technique. Still, managing volume status in HF patients with CKD requires a multidisciplinary approach involving cardiologists and nephrologists to optimize treatment and improve outcomes. The issue is further complicated by the absence of a gold standard to quantify lung or tissue extravascular water accumulation accurately. Ultrafiltration during hemodialysis effectively controls lung congestion in kidney failure patients on chronic dialysis [15]. In HF patients with residual kidney function, ultrafiltration devices permit fluid removal but with conflicting results regarding safety and efficacy [16]. Peritoneal dialysis (PD) is a proposed therapy for refractory HF with fluid overload, offering continuous ultrafiltration and potentially leading to reduced hospitalization [17]. However, evidence supporting its efficacy is primarily based on small observational studies, with a notable lack of randomized controlled trials (RCTs) [18]. While some studies suggest that PD improves symptoms and functional status, definitive conclusions are limited. RCTs to establish role, optimal timing, and patient selection of PD in HF management are a public health and research priority. At the moment, without robust trials, PD remains an experimental and empiric approach rather than a standard therapy for HF patients with diuretic resistance.

### Sodium restriction (Table 3)

The use of a strict low-sodium diet (<1.5 g/day according to the AHA/ACC [7] or <2.0 g/day according to the World Health Organization) in HF is controversial. Mortality and the risk for hospitalization are not reduced with strict sodium restriction in patients with HF [19], although some symptomatic improvement may be expected. A sodium intake <2 g/day is recommended in the 2024 KDIGO CKD guidelines [20]. However, this target is hard to achieve in clinical practice [21]. In a study based on the Chronic Renal Insufficiency Cohort that accurately measured sodium intake by applying at least three 24 h urine collections, the relationship between sodium intake and the risk of cardiovascular events flattened out below 130–150 mmol sodium/day [22], corresponding to ∼3 g/day, suggesting that this level may be assumed to be a safe and less strict target in CKD and HF. In the Study of Dietary Intervention under 100 mmol in Heart Failure (SODIUM-HF) trial [23] reduction of sodium intake to <100 mmol/day in patients with HF failed to reduce mortality and risk of hospitalization.

### Diuretics (Table 3)

Diuretics are the cornerstone of management in HF [7], but there are challenges to optimize their use in many instances. Loop diuretics increase sympathetic nervous system activity in patients with reduced left ventricular function and may adversely affect clinical outcomes [24], thus questioning the value of these agents in the long term and in high doses. Diuretics can lead to electrolyte imbalances and can worsen renal function, requiring frequent monitoring and dose adjustments. This is particularly important in patients with CKD, where the balance between efficacy and safety becomes even more delicate. Thiazide diuretics have long been considered of scarce efficacy in stage 4 CKD. However, a systematic review appraising randomized trials in this population [20] showed that these drugs may be helpful even in patients with advanced CKD, providing sequential nephron target inhibition [25]. Thiazides cause a negative sodium balance, reduce body fluids 1–2 l and improve hypertension control. In the Combination of Loop with Thiazide-type Diuretics in Patients with Decompensated Heart Failure (CLOROTIC) study—a trial in patients with an average estimated glomerular filtration rate (GFR) of 40 ml/min/1.73 m^2^—the addition of hydrochlorothiazide to loop diuretics was associated with a more significant weight loss and increase in urine output than with loop diuretics only [26]. In the Chlorthalidone in Chronic Kidney Disease trial, this diuretic reduced 24 h ambulatory blood pressure (BP) by 11 mmHg in patients with advanced CKD [27]. In the Antihypertensive and Lipid‐Lowering Treatment to Prevent Heart Attack Trial this drug reduced the risk for HF compared with other antihypertensive treatments (amlodipine, lisinopril, and doxazosin) [28]. In another trial in HF patients studying loop diuretic resistance, dapagliflozin and metolazone were compared, and the two drugs were equally effective in relieving congestion. A potential advantage of those assigned to dapagliflozin was a lower incidence of metabolic alterations; still, this effect did not affect length of stay or mortality [29]. In principle, these drugs are not necessarily alternative and may be all combined to relieve congestion. Carbonic anhydrase inhibitors in combination with loop diuretics may overcome diuretic resistance by blocking the proximal tubule compensatory response to loop diuretics. In the Acetazolamide in Decompensated Heart Failure with Volume Overload trial [30] the addition of acetazolamide—a carbonic anhydrase inhibitor—to loop diuretic therapy resulted in a greater incidence of successful decongestion. In this trial there was a significant interaction between the effect of acetazolamide and baseline renal function, the drug being more effective in patients with eGFR ≤40 ml/min/1.73 m^2^. However, this greater efficacy on decongestion is apparently associated with a greater risk of worsening renal function.

### Mineralocorticoid receptor antagonists (Table 3)

MRAs are recommended for HF, particularly in patients with HFrEF [31]. Traditional MRAs, such as spironolactone and eplerenone, have been extensively used and are supported by solid evidence from large, randomized trials. However, their use is often limited by hyperkalemia and renal dysfunction. Newer MRAs, such as finerenone, have emerged with a more favorable safety profile and a lower risk of hyperkalemia. Finerenone improves kidney and cardiovascular outcomes in patients with CKD and type 2 diabetes and is recommended by the 2024 KDIGO guidelines [7, 20] in this patient population. Hyponatremia, hypokalemia, dehydration, and acute kidney injury are side effects that demand a vigilant attitude by physicians prescribing these drugs.

As discussed before, combining different diuretics, such as loop diuretics with thiazide-type diuretics or carbonic anhydrase inhibitors (sequential nephron target inhibition), can be effective in managing fluid overload in HF [25]. However, combination of these drugs also increases the risk of electrolyte imbalances and further renal impairment, underscoring the need to regularly assess renal function and electrolytes, especially for the risk of hypokalemia (see next). Introducing novel therapies such as neprilysin inhibitors [25] adds another layer of complexity. These agents, while beneficial, require careful consideration of renal function due to their impact on renal hemodynamics and electrolyte balance.

By managing a patient's hydration status, one can obtain a more accurate measure of patients’ true GFR. Even mild degrees of dehydration can transiently reduce the eGFR, while overhydration can have the opposite effect. Differentiating between transient, reversible pre-renal causes of eGFR reductions and stable kidney dysfunction is of obvious relevance in the management of patients with concomitant HF and CKD [32].

### Electrolyte imbalances (Table 3)

Hyperkalemia and hypokalemia are both consequences of aggressive therapy for HF; the former is attributable in part to RAAS blockade and MRA use [33], and the latter to potent diuretic use. Both can lead to dysrhythmias in vulnerable individuals. Data are poor supporting the use of potassium binders to reduce events, although they effectively lower potassium levels. Hypokalemia requires potassium supplementation and adjustments in diuretic therapy by adding MRAs. The risk of hypokalemia is reduced as kidney dysfunction progresses, while the opposite risk of hyperkalemia ensues.

Hyponatremia, an alteration secondary to an increase in anti-diuretic hormone levels, can be multifactorial. The main factor underlying this alteration is extracellular volume contraction triggered by the use of diuretics in conjunction with unrestricted water intake. Hyponatremia has a prevalence ranging between 11% and 27% in HF patients [34] and is associated with higher morbidity and mortality [35–37]. Treatment involves fluid restriction and careful adjustment of diuretic therapy to balance sodium and fluid levels. Recent protocols for “diuretic resistance” and other agents (such as tolvaptan and SGLT2i), have improved the management of hyponatremia, especially in hospitalized patients [38].

### Abnormalities of mineral metabolism, vascular disease, and heart failure (Table 3)

In patients with CKD and HF, the prevalence of serum calcium alterations can be as high as 40% to 60% in stages G4–5 CKD patients, mainly when HF is also present. In the Danish national registries [39], among patients with chronic HF, 33% had hypocalcemia, and 4% had hypercalcemia. Both hypo- and hypercalcemia predict high short- and long-term mortality, independent of other factors.

Serum phosphate levels mainly increase in advanced (stages G4 and G5) CKD. Hyperphosphatemia in these patients is associated with cardiac hypertrophy, which may worsen cardiac contractility and HF [40]. Calcium and phosphate metabolism alterations are the hallmarks of the CKD-bone mineral disorder (CKD-BMD) and cause vascular and cardiac calcifications. Calcific aortic stenosis (CAS) is classified into mild, moderate, and severe based on echocardiographic and computed tomography criteria [41]. Aorti stenosis severity is determined by aortic valve area (mild >1.5 cm², moderate 1.0–1.5 cm², severe <1.0 cm²), mean gradient (mild <20 mmHg, moderate 20–39 mmHg, severe ≥40 mmHg), peak velocity (mild <3.0 m/s, moderate 3.0–3.9 m/s, severe ≥4.0 m/s), and calcium score (severe if >2000 in men or >1200 in women). Risk factors and therapeutic targets for more severe stenosis include advanced age, male sex, hypertension, hyperlipidemia, diabetes, CKD, bicuspid aortic valve, smoking, and inflammatory diseases [42]. These factors contribute to faster progression and increased calcification of the valve. The prevalence of CAS in CKD patients is 27% or higher [43]. CAS increases afterload, generating or aggravating left ventricular hypertrophy (LVH). Vascular calcification can lead to stiffer vessels, increasing pulse wave velocity, thus increasing left ventricular end-diastolic pressure, which may exacerbate HF. There are now multiple options for valve replacement, including surgery and transcatheter aortic valve implantation, for which timing and coordination with cardiologists are important. Options will depend on patient characteristics and healthcare system organization [43].

In managing mineral and bone disorders in CKD, phosphate binders are recommended when hyperphosphatemia becomes apparent, typically in CKD stages G3–5. Calcium-based binders are used initially but should be minimized if serum calcium levels are high [44]. Calcitriol or its analogs, such as paricalcitol and doxercalciferol, are commonly recommended starting from CKD stage G3. Calcimimetics are primarily used in later stages, particularly stage G5, to manage secondary hyperparathyroidism by lowering parathyroid hormone levels without increasing serum calcium or phosphate. In dialysis patients, treatment needs to be more aggressive due to the severity of the condition, with regular monitoring and adjustments essential to prevent complications such as vascular calcification and bone disease progression. Vitamin K antagonists, such as warfarin, used for thrombosis prevention in kidney failure patients with atrial fibrillation, can increase the risk of vascular calcification. Conversely, vitamin K supplementation has been suggested to prevent vascular calcification through anti-inflammatory mechanisms. No randomized trial targeting vascular calcification or associated biomarkers has shown favorable effects on major clinical outcomes in CKD patients. Arterial stiffness, related to vascular calcification, is increased in CKD and HF, predicting cardiovascular events independent of BP [35, 36, 45]. Methods for measuring this parameter and calcium accumulation in the vessel walls are discussed in detail in a separate review [46]. Factors impacting on arterial stiffness are largely the same that affect arterial BP [47]. Targeting arterial stiffness per se remains an unmet high-priority therapeutic target to lessen the global burden of cardiovascular disease.

Hypomagnesemia can occur due to diuretic use and is associated with increased risk of arrhythmias, including atrial fibrillation, and sudden death. Magnesium supplementation may be necessary to maintain normal levels and prevent complications [48]. Depending on the clinical context, appropriate adjustments in medication regimens, dietary modifications, and supplements should be applied to manage these imbalances.

### Anticoagulation in atrial fibrillation (Table 4)

In patients with HF and CKD, atrial fibrillation (AF) is common, and significantly increases the risk of thromboembolic events. Anticoagulation therapy would be essential to mitigate this risk, yet it presents unique challenges in the context of advanced CKD. Vitamin K antagonists (VKAs), such as warfarin, acenocoumarol, phenprocoumon, and phenindione, have been the traditional choice for anticoagulation because their activity can be monitored and adjusted with the International Normalized Ratio. However, their use in CKD is complicated by the need for regular monitoring, the increased risk of bleeding due to impaired renal function and the absence of clear documentation of efficacy [49]. Metabolism and clearance of VKAs are affected by renal and hepatic impairment, necessitating frequent dose adjustments and close monitoring to maintain therapeutic levels without increasing bleeding risk. No study has so far shown benefits on cardioembolic stroke or other major adverse cardiovascular events in patients with stage G4 or G5 CKD. The direct oral anticoagulants (DOACs) dabigatran, rivaroxaban, apixaban, and edoxaban offer an alternative to VKAs, with the advantage of fixed dosing and no routine coagulation monitoring. However, their use in patients with advanced CKD (eGFR <30 ml/min/1.73 m²) is limited due to concern about drug accumulation and bleeding [50]. Apixaban is often preferred in this population due to its relatively favorable safety profile and its lowest renal clearance, but even this drug requires careful consideration of renal function because of poor evidence of better efficacy or safety compared to VKAs in patients with end-stage renal disease [51]. The decision to the off-label use of DOACs must thus balance the risk of stroke against the potential for bleeding, taking into account individual patient factors such as age, comorbidities, and concomitant medications.

### Management of hypertension, diabetes, and hyperlipidemia (Table 4)

Hypertension is common in patients with HF and CKD, and its management is critical to prevent further cardiovascular and renal deterioration. Determining optimal BP targets in this population is challenging, particularly in patients with high arterial stiffness and vascular calcification, conditions increasingly prevalent across progressively lower eGFR strata [52]. There is no specific clinical trial testing targeting BP reductions on outcomes in patients with HF and concomitant hypertension. The 2022 ACC/AHA and the 2021 ESC guidelines for HF recommend a BP target <130 mmHg both in patients with HFrEF and HF with preserved EF (HFpEF) [8]. The ESC guidelines remark that uncontrolled hypertension is rare in patients receiving optimal treatment for HF, namely beta-blockers, ACE inhibitors, angiotensin receptor-neprilysin inhibitors, SGLT2 inhibitors and MRAs [53]. The prescription of these drugs in relationship to renal function is illustrated in Table 2. In addition, ESC guidelines suggest that, if further BP lowering is required in patients with no signs of fluid retention, amlodipine and felodipine are safe options [43].

Glycemic control is crucial to prevent microvascular and possibly also macrovascular complications. The KDIGO 2022 guidelines on diabetes in CKD offer detailed recommendations for the use of oral antidiabetic drugs in patients with CKD, emphasizing the importance of adjusting treatment based on kidney function. These recommendations are of obvious relevance also for HF patients with CKD. The SGLT2i empagliflozin and dapaglifozin are recommended for adults with type 2 diabetes and CKD with an eGFR ≥20 and ≥25 ml/min per 1.73 m², respectively, independent of diabetes. These drugs should be continued even if the eGFR falls below this threshold unless the patient cannot tolerate them or has to start kidney replacement therapy [54]. For prescription of canagliflozin and ertugliflozin the eGFR should be ≥30 and ≥45 ml/min/1.73 m^2^, respectively. In the same guidelines glucagon-like peptide-1 receptor agonists (GLP-1 RA) are suggested for patients who have not achieved glycemic targets with metformin and SGLT2 inhibitors or cannot use these medications. The selection of specific GLP-1 RAs with proven cardiovascular benefits (e.g. semaglutide, tirzepatide) is recommended given the high risk of cardiovascular events in patients with CKD and HF [55, 56]. Metformin remains a cornerstone of diabetes management but requires dose reduction when eGFR drops below 45 ml/min per 1.73 m² and should be discontinued for eGFR <30 ml/min per 1.73 m² due to the risk of lactic acidosis.

For patients with elevated LDL-C, statins and ezetimibe are recommended to reduce cardiovascular risk in CKD [57]. However, the clinical benefits of LDL-C lowering in patients with advanced CKD or on dialysis are less clear and treatment decisions should be individualized, considering the patients’ overall cardiovascular risk profile and life expectancy. For patients with HF, particularly those with reduced ejection fraction, the role of statins is also controversial [58]. While statins are not routinely recommended solely for HF management, they may be beneficial in patients with concomitant atherosclerotic cardiovascular disease. The use of other lipid-lowering agents, such as proprotein convertase subtilisin/kexin type 9 inhibitors and bempedoic acid, may be considered in specific cases where statin/ezetimibe therapy is insufficient or not tolerated [58, 59].

### Inflammation (Table 5)

HF is characterized by an increase in major pro-inflammatory cytokines, such as tumor necrosis factor α and interleukin-6 (IL-6), and such an increase predicts mortality in this condition [60]. Chronic inflammation is a hallmark of CKD [61], triggers vascular calcification and arterial stiffness [62] and plays a critical role in renal and cardiovascular disease progression. Guideline-directed medical therapies for HF have been shown to reduce cytokine levels, and their benefit in HF and CKD may at least in part be mediated by anti-inflammatory effects [63]. Whether treatments designed to reduce inflammation specifically are beneficial remains an area of active investigation [64].

### Anemia (Table 5)

The use of intravenous iron in HF patients with demonstrated iron deficiency, even subclinical and irrespective of Hb, appears to improve cardiac function in HF trials. In the Proactive IV Iron Therapy in Hemodialysis Patients (PIVOTAL) trial [65], a high-dose intravenous iron regimen reduced the risk for the primary endpoint, a composite of nonfatal myocardial infarction, nonfatal stroke, hospitalization for HF, or death by 15% compared to a low-dose regimen administered reactively, and resulted in lower doses of erythropoiesis-stimulating agents being administered. Iron metabolism metrics, mostly based on transferrin saturation, should be included in the laboratory evaluation of patients diagnosed with HF to timely identify patients in whom intravenous iron replacement should be enacted.

### Proteinuria in heart failure (Table 5)

Proteinuria reflects glomerular injury, which can exacerbate HF by contributing to fluid retention and increased cardiovascular risk. Hemodynamic factors that contribute to proteinuria in HF include elevated central venous pressure, reduced renal perfusion, and vascular congestion, which impair glomerular filtration. These changes lead to increased glomerular permeability and protein leakage into urine [66]. Treating proteinuria in HF involves addressing underlying causes, such as hypertension and diabetes, as well as using medications such as ACE inhibitors or angiotensin receptor blockers, which can reduce proteinuria and provide renal protection. Because of its association with worse outcomes, proteinuria should indeed be considered a treatment target in HF management to improve both renal and cardiovascular prognosis. The use of RAAS inhibitors must be, however, balanced with the risk of hyperkalemia and worsening renal function, particularly in advanced CKD. SGLT2 inhibitors reduce proteinuria and reduce the risk for HF in CKD patients [44].

### New technologies and new opportunities for prevention

The current care of patients with HF and CKD involves regular monitoring, medication management, lifestyle changes, and the collaboration of cardiologists and nephrologists. However, this traditional model is often fragmented, and there appears to be the need for it to be more cohesive and efficient. There is a need to re-think care delivery models, and how to integrate new technologies to improve outcomes. Telemedicine and remote monitoring are transforming patient care by enabling real-time consultations and continuous health tracking, regardless of a patient's location. These tools allow healthcare providers to monitor vital signs, fluid status, and other critical health parameters, facilitating early detection and intervention for issues such as fluid retention and cardiac decompensation. In addition to these non-invasive methods, devices such as the Cardiac Micro Electro Mechanical System, a small, implantable device that monitors pulmonary artery pressure providing valuable data to help manage HF, offer the possibility of continuous invasive monitoring by measuring pulmonary artery pressure directly. This technology provides valuable data that can guide treatment adjustments, helping to prevent hospitalizations and improve patient outcomes, especially in those at high risk of decompensation. Furthermore, advanced HF therapies, including ventricular assistance devices and heart transplantation, offer life-saving options for patients with severe HF. These interventions can significantly improve quality of life and survival rates of eligible patients. Advances in electrophysiology are also crucial in HF management. Procedures such as ablation of AF or ventricular tachycardia and biventricular pacing are recommended in current HF guidelines and also apply to appropriate patients with HF and advanced kidney disease. These techniques help maintain rhythm control and improve cardiac function, reducing symptoms and enhancing patients’ well-being.

## FUTURE DIRECTIONS

In this era of “big data,” including use of very large databases from health records, registries, and multicenter research studies, there is an opportunity to learn more about for both characterization and understanding of CKD and HF, as well as for optimizing the use of the pillars of therapy for HF: beta-blockers, ACE inhibitors, angiotensin II receptor inhibitors, angiotensin receptor-neprilysin inhibitors, MRAs, and SGLT2 inhibitors.

Artificial intelligence (AI) and machine learning are also poised to transform personalized care. AI-based algorithms applied to standard clinical data, such as laboratory results, medication adherence, and lifestyle factors, or even the standard electrocardiogram, can identify specific profiles and patterns, and predict which patients are at higher risk for complications and hospitalization. This predictive capability allows for proactive interventions, including quick lifestyle modifications or preemptive medication adjustments.

Multiple trials have now adopted combined cardiovascular and kidney endpoints, and methodological advances have been made about their use [67]. Because many of these agents for HF are also beneficial for CKD, we should commit to monitoring and reviewing all new agents, both in combination and alone, for their impact on important patient outcomes.

Novel anti-inflammatory agents, such as the IL-1β antagonist canakinumab and the IL-6 antagonist ziltivekimab, are powerful tools for countering inflammation. Still, these drugs have not yet been tested purely in HF with coexisting kidney disease. However, one ongoing trial in patients with mildly reduced or preserved ejection fraction will also inform about the progression of kidney disease [68].

Looking ahead, advances in omics sciences offer unprecedented opportunities for personalization. Genetic testing can already identify specific markers predicting patient response to HF medications [69] Omics data on HF can inform the development of novel treatments or innovative treatment strategies for this disease. Multiomics can more efficiently identify potential treatment targets for further study. Yet, there are several barriers that must be overcome to maximize the utility of these promising studies.

## CONCLUSION

Up to one-third of patients with CKD also suffer from HF, presenting both a significant healthcare burden and a valuable opportunity for advancing patient outcomes. This dual diagnosis complicates treatment due to the interrelated pathophysiology of the heart and kidneys, necessitating a comprehensive approach to better care. Recent developments in HF therapies, tested across diverse populations—including those with diabetes mellitus and varying stages of CKD—provide new avenues for addressing this complex patient group. Personalizing treatment involves optimizing timing, dosage, and type of medications tailored to individual patient needs. This is facilitated by newer models of collaborative care, which integrate multidisciplinary teams to manage the multifaceted aspects of HF and CKD. By integrating novel therapies and technologies within a collaborative care framework, healthcare providers can offer more effective and individualized treatment strategies, ultimately improving quality of life for patients with coexisting HF and CKD. Enhanced communication and coordination among healthcare providers will ensure comprehensive care that addresses both cardiovascular and renal aspects of these conditions.

## Data Availability

No new data were generated or analyzed in support of this research.

## References

[1] Ru SC, Lv SB, Li ZJ. Incidence, mortality, and predictors of acute kidney injury in patients with heart failure: a systematic review. ESC Heart Failure 2023; 10:3237–49. 10.1002/ehf2.14520 37705352 PMC10682870

[2] Roth GA, Abate D, Hassen Abate K et al. Global, regional, and national age-sex-specific mortality for 282 causes of death in 195 countries and territories, 1980-2017: a systematic analysis for the Global Burden of Disease Study 2017 GBD 2017 Causes of Death Collaborators*. Lancet 2018; 392:1736–88. https://vizhub.health 30496103 10.1016/S0140-6736(18)32203-7 PMC6227606

[3] Heywood JT, Fonarow GC, Costanzo MR et al. High prevalence of renal dysfunction and its impact on outcome in 118,465 patients hospitalized with acute decompensated heart failure: a report from the ADHERE database. J Card Fail 2007; 13:422–30. https://pubmed.ncbi.nlm.nih.gov/17675055 17675055 10.1016/j.cardfail.2007.03.011

[4] Lesyuk W, Kriza C, Kolominsky-Rabas P. Cost-of-illness studies in heart failure: a systematic review 2004-2016. BMC Cardiovasc Disord 2018; 18:1–11. 10.1186/s12872-018-0815-3 29716540 PMC5930493

[5] Matsushita K, van der Velde M, Astor BC et al. Association of estimated glomerular filtration rate and albuminuria with all-cause and cardiovascular mortality: a collaborative meta-analysis of general population cohorts. Lancet 2010; 375:2073–81. https://pmc.ncbi.nlm.nih.gov/articles/PMC3993088/20483451 10.1016/S0140-6736(10)60674-5 PMC3993088

[6] Halimi J-M, de Fréminville J-B, Gatault P et al. Long-term impact of cardiorenal syndromes on major outcomes based on their chronology: a comprehensive French nationwide cohort study. Nephrol Dial Transplant 2022; 37:2386–97. http://www.ncbi.nlm.nih.gov/pubmed/35438794 35438794 10.1093/ndt/gfac153

[7] Heidenreich PA, Bozkurt B, Aguilar D et al. 2022 AHA/ACC/HFSA Guideline for the management of Heart Failure: a report of the American College of Cardiology/American Heart Association Joint Committee on Clinical Practice Guidelines. Circulation 2022; 145:E895–E1032. Available from: https://pubmed.ncbi.nlm.nih.gov/35363499/35363499 10.1161/CIR.0000000000001063

[8] McDonagh TA, Metra M, Adamo M et al. 2021 ESC guidelines for the diagnosis and treatment of acute and chronic heart failure. Eur Heart J 2021; 42:3599–726. 10.1093/eurheartj/ehab368 34447992

[9] Le D, Grams ME, Coresh J et al. Sacubitril-Valsartan in patients requiring hemodialysis. JAMA Netw Open 2024; 7:e2429237. Available from: https://jamanetwork.com/journals/jamanetworkopen/fullarticle/2822448 39163041 10.1001/jamanetworkopen.2024.29237 PMC11337068

[10] Ku E, Tighiouart H, Mcculloch CE et al. Association between acute declines in eGFR during renin–angiotensin system inhibition and risk of adverse outcomes. J Am Soc Nephrol 2024; 35. https://pubmed.ncbi.nlm.nih.gov/38889197/10.1681/ASN.0000000000000426 PMC11452131 38889197

[11] Hsiao FC, Lin CP, Yu CC et al. Angiotensin receptor-neprilysin inhibitors in patients with heart failure with reduced ejection fraction and advanced chronic kidney disease: a retrospective multi-institutional study. Front Cardiovasc Med 2022; 9:794707. 35360037 10.3389/fcvm.2022.794707 PMC8963957

[12] Kraus BJ, Weir MR, Bakris GL et al. Characterization and implications of the initial estimated glomerular filtration rate “dip” upon sodium-glucose cotransporter-2 inhibition with empagliflozin in the EMPA-REG OUTCOME trial. Kidney Int 2021; 99:750–62. https://pubmed.ncbi.nlm.nih.gov/33181154/33181154 10.1016/j.kint.2020.10.031

[13] Moissl UM, Wabel P, Chamney PW et al. Body fluid volume determination via body composition spectroscopy in health and disease. Physiol Meas 2006; 27:921–33. 10.1088/0967-3334/27/9/012 16868355

[14] Gargani L, Girerd N, Platz E et al. Lung ultrasound in acute and chronic heart failure: a clinical consensus statement of the European Association of Cardiovascular Imaging (EACVI). Eur Heart J Cardiovasc Imag 2023; 24:1569–82. https://pubmed.ncbi.nlm.nih.gov/37450604/10.1093/ehjci/jead169 PMC11032195 37450604

[15] Zoccali C, Torino C, Mallamaci F et al. A randomized multicenter trial on a lung ultrasound-guided treatment strategy in patients on chronic hemodialysis with high cardiovascular risk. Kidney Int 2021; 100:1325–33. http://www.ncbi.nlm.nih.gov/pubmed/34418415 34418415 10.1016/j.kint.2021.07.024

[16] Costanzo MR, Ronco C, Abraham WT et al. Extracorporeal ultrafiltration for fluid overload in heart failure: current status and prospects for further research. J Am Coll Cardiol 2017; 69:2428. https://pmc.ncbi.nlm.nih.gov/articles/PMC5632523/28494980 10.1016/j.jacc.2017.03.528 PMC5632523

[17] François K, Ronco C, Bargman JM. Peritoneal dialysis for chronic congestive heart failure. Blood Purif 2015; 40:45–52. 10.1159/000430084 26111872

[18] Morales RO, Barbosa F, Farre N. Peritoneal dialysis in heart failure: focus on kidney and ventricular dysfunction. Rev Cardiovasc Med 2021; 3:649–57. 10.31083/j.rcm2203075 34565067

[19] Urban S, Fułek M, Błaziak M et al. Role of dietary sodium restriction in chronic heart failure: systematic review and meta-analysis. Clin Res Cardiol 2024; 113:1331–42. 10.1007/s00392-023-02256-7 37389661 PMC11371846

[20] Stevens PE, Ahmed SB, Carrero JJ et al. KDIGO 2024 clinical practice guideline for the evaluation and management of chronic kidney disease. Kidney Int 2024; 105:S117–S314. http://www.kidney-international.org/article/S0085253823007664/fulltext 38490803 10.1016/j.kint.2023.10.018

[21] Wang AYM, Mallamaci F, Zoccali C. What is central to renal nutrition: protein or sodium intake? Clin Kidney J 2023; 16:1824–33. https://pubmed.ncbi.nlm.nih.gov/37915942/37915942 10.1093/ckj/sfad151 PMC10616450

[22] Mills KT, Chen J, Yang W et al. Sodium excretion and the risk of cardiovascular disease in patients with chronic kidney disease. JAMA 2016; 315:2200–10. https://pubmed.ncbi.nlm.nih.gov/27218629/27218629 10.1001/jama.2016.4447 PMC5087595

[23] Ezekowitz JA, Colin-Ramirez E, Ross H et al. Reduction of dietary sodium to less than 100 mmol in heart failure (SODIUM-HF): an international, open-label, randomised, controlled trial. Lancet 2022; 399:1391–400. https://www.thelancet.com/action/showFullText?pii=S0140673622003695 35381194 10.1016/S0140-6736(22)00369-5

[24] Onitsuka H, Koyama S, Ideguchi T et al. Impact of short-acting loop diuretic doses and cardiac sympathetic nerve abnormalities on outcomes of patients with reduced left ventricular function. Medicine (Baltimore) 2019; 98:e14657. 30813209 10.1097/MD.0000000000014657 PMC6407956

[25] Gogikar A, Nanda A, Janga LSN et al. Combination diuretic therapy with thiazides: a systematic review on the beneficial approach to overcome refractory fluid overload in heart failure. Cureus 2023; 15:e44624. https://pmc.ncbi.nlm.nih.gov/articles/PMC10500380/37720125 10.7759/cureus.44624 PMC10500380

[26] Trullàs JC, Morales-Rull JL, Casado J et al. Combining loop with thiazide diuretics for decompensated heart failure: the CLOROTIC trial. Eur Heart J 2023; 44:411–21. 10.1093/eurheartj/ehac689 36423214

[27] Agarwal R, Sinha AD, Cramer AE et al. Chlorthalidone for hypertension in advanced chronic kidney disease. N Engl J Med 2021; 385:2507–19. https://www.nejm.org/doi/full/10.1056/NEJMoa2110730 34739197 10.1056/NEJMoa2110730 PMC9119310

[28] Davis BR, Piller LB, Cutler JA et al. Role of diuretics in the prevention of heart failure: the antihypertensive and lipid-lowering treatment to prevent heart attack trial. Circulation 2006; 113:2201–10. https://pubmed.ncbi.nlm.nih.gov/16651474/16651474 10.1161/CIRCULATIONAHA.105.544031

[29] Yeoh SE, Osmanska J, Petrie MC et al. Dapagliflozin vs. metolazone in heart failure resistant to loop diuretics. Eur Heart J 2023; 44:2966–77. https://pubmed.ncbi.nlm.nih.gov/37210742/37210742 10.1093/eurheartj/ehad341 PMC10424881

[30] Mullens W, Dauw J, Martens P et al. Acetazolamide in acute decompensated heart failure with volume overload. N Engl J Med 2022; 387:1185–95. https://pubmed.ncbi.nlm.nih.gov/36027559/36027559 10.1056/NEJMoa2203094

[31] McDonagh TA, Metra M, Adamo M et al. 2023 Focused update of the 2021 ESC Guidelines for the diagnosis and treatment of acute and chronic heart failure: developed by the task force for the diagnosis and treatment of acute and chronic heart failure of the European Society of Cardiology (ESC) with the special contribution of the Heart Failure Association (HFA) of the ESC. Eur J Heart Fail 2024; 26:5–17. https://pubmed.ncbi.nlm.nih.gov/38169072/38169072 10.1002/ejhf.3024

[32] Mullens W, Damman K, Testani JM et al. Evaluation of kidney function throughout the heart failure trajectory—a position statement from the Heart Failure Association of the European Society of Cardiology. Eur J Heart Fail 2020; 22:584–603. 10.1002/ejhf.1697 31908120

[33] Weinstein J, Girard LP, Lepage S et al. Prevention and management of hyperkalemia in patients treated with renin–angiotensin–aldosterone system inhibitors. Can Med Assoc J 2021; 193:E1836–E41. https://pmc.ncbi.nlm.nih.gov/articles/PMC8648362/34872955 10.1503/cmaj.210831 PMC8648362

[34] Zhao W, Qin J, Lu G et al. Association between hyponatremia and adverse clinical outcomes of heart failure: current evidence based on a systematic review and meta-analysis. Front Cardiovasc Med 2023; 10:1339203. 10.3389/fcvm.2023.1339203 38204798 PMC10777843

[35] Dewolfe A, Lopez B, Arcement LM et al. Low serum sodium as a poor prognostic indicator for mortality in congestive heart failure patients. Clin Cardiol 2010; 33:E13–E7. https://pubmed.ncbi.nlm.nih.gov/21184540/10.1002/clc.20560 PMC6653197 21184540

[36] Klein L, O'Connor CM, Leimberger JD et al. Lower serum sodium is associated with increased short-term mortality in hospitalized patients with worsening heart failure: results from the outcomes of a prospective trial of intravenous milrinone for exacerbations of chronic heart failure (OPTIME-CHF) study. Circulation 2005; 111:2454–60. https://pubmed.ncbi.nlm.nih.gov/15867182/15867182 10.1161/01.CIR.0000165065.82609.3D

[37] Romanovsky A, Bagshaw S, Rosner MH. Hyponatremia and congestive heart failure: a marker of increased mortality and a target for therapy. Int J Nephrol 2011; 2011:1–7. https://pubmed.ncbi.nlm.nih.gov/21603106/10.4061/2011/732746 PMC3097052 21603106

[38] Cox ZL, Hung R, Lenihan DJ et al. Diuretic strategies for loop Diuretic resistance in acute heart failure: the 3T trial. JACC: Heart Fail 2020; 8:157–68. 31838029 10.1016/j.jchf.2019.09.012 PMC7058489

[39] Jensen ASC, Polcwiartek C, Søgaard P et al. The association between serum calcium levels and short-term mortality in patients with chronic heart failure. Am J Med 2019; 132:200–8. 10.1016/j.amjmed.2018.10.006 30691552

[40] Christopoulou EC, Filippatos TD, Megapanou E et al. Phosphate imbalance in patients with heart failure. Heart Fail Rev 2017; 22:349–56. https://pubmed.ncbi.nlm.nih.gov/28432604/28432604 10.1007/s10741-017-9615-6

[41] Otto CM, Nishimura RA, Bonow RO et al. 2020 ACC/AHA Guideline for the management of patients with valvular Heart disease: a report of the American College of Cardiology/American Heart Association Joint Committee on Clinical Practice Guidelines. Circulation 2021; 143:E72–E227. 10.1161/CIR.0000000000000923 33332150

[42] Wu C-F, Liu P-Y, Wu T-J et al. Therapeutic modification of arterial stiffness: an update and comprehensive review. World J Cardiol 2015; 7:742. https://pmc.ncbi.nlm.nih.gov/articles/PMC4660469/26635922 10.4330/wjc.v7.i11.742 PMC4660469

[43] Hutcheson JD, Goettsch C. Cardiovascular calcification heterogeneity in chronic kidney disease. Circ Res 2023; 132:993–1012. 10.1161/CIRCRESAHA.123.321760 37053279 PMC10097496

[44] KDIGO 2017 clinical practice guideline update for the diagnosis, evaluation, prevention, and treatment of chronic kidney disease–Mineral and bone disorder (CKD-MBD). Kidney Int Suppl 2017; 7:1–59. 10.1016/j.kisu.2017.04.001 PMC6340919 30675420

[45] Chirinos JA, Segers P, Hughes T et al. Large-artery stiffness in health and disease: JACC State-of-the-Art Review. J Am Coll Cardiol 2019; 74:1237–63. 10.1016/j.jacc.2019.07.012 31466622 PMC6719727

[46] Zoccali C, Mark PB, Sarafidis P et al. Diagnosis of cardiovascular disease in patients with chronic kidney disease. Nat Rev Nephrol 2023; 19:733–46. https://www.nature.com/articles/s41581-023-00747-4 37612381 10.1038/s41581-023-00747-4

[47] Kim HL. Arterial stiffness and hypertension. Clin Hypertens 2023; 29:31. https://pmc.ncbi.nlm.nih.gov/articles/PMC10691097/38037153 10.1186/s40885-023-00258-1 PMC10691097

[48] Vermeulen EA, Vervloet MG. Magnesium administration in chronic kidney disease. Nutrients 2023; 15:547. 10.3390/nu15030547 36771254 PMC9920010

[49] Cases A, Gomez P, Broseta JJ et al. Non-valvular atrial fibrillation in CKD: role of vitamin K antagonists and direct oral anticoagulants. A narrative review. Front Med 2021; 8:654620. 10.3389/fmed.2021.654620 PMC8484537 34604247

[50] Chen A, Stecker E, Warden BA. Direct oral anticoagulant use: a practical guide to common clinical challenges. J Am Heart Assoc 2020; 9:e017559. 10.1161/JAHA.120.017559 32538234 PMC7670541

[51] Mandt SR, Thadathil N, Klem C et al. Apixaban use in patients with Kidney impairment: a review of pharmacokinetic, interventional, and observational study data. Am J Cardiovasc Drugs 2024; 24:603–24. https://pubmed.ncbi.nlm.nih.gov/39102124/39102124 10.1007/s40256-024-00664-2 PMC11344734

[52] Budoff MJ, Rader DJ, Reilly MP et al. Relationship of estimated GFR and coronary artery calcification in the CRIC (Chronic Renal Insufficiency Cohort) study. Am J Kidney Dis 2011; 58:519–26. 10.1053/j.ajkd.2011.04.024 21783289 PMC3183168

[53] Visseren FLJ, Mach F, Smulders YM et al. 2021 ESC guidelines on cardiovascular disease prevention in clinical practice. Eur Heart J 2021; 42:3227–337. http://www.ncbi.nlm.nih.gov/pubmed/34458905 34458905 10.1093/eurheartj/ehab484

[54] KDIGO 2022 clinical practice guideline for diabetes management in chronic kidney disease. Kidney Int 2022; 102:S1–S127. https://pubmed.ncbi.nlm.nih.gov/36272764/36272764 10.1016/j.kint.2022.06.008

[55] Kosiborod MN, Abildstrøm SZ, Borlaug BA et al. Semaglutide in patients with heart failure with preserved ejection fraction and obesity. N Engl J Med 2023; 389:1069–84. 10.1056/NEJMoa2306963 37622681

[56] Packer M, Zile MR, Kramer CM et al. Tirzepatide for heart failure with preserved ejection fraction and obesity. N Engl J Med 2025; 392:427–37. https://pubmed.ncbi.nlm.nih.gov/39555826/39555826 10.1056/NEJMoa2410027

[57] Baigent C, Landray MJ, Reith C et al. The effects of lowering LDL cholesterol with simvastatin plus ezetimibe in patients with chronic kidney disease (Study of Heart and Renal Protection): a randomised placebo-controlled trial. Lancet 2011; 377:2181–92. 10.1016/S0140-6736(11)60739-3 21663949 PMC3145073

[58] Lee MMY, Sattar N, McMurray JJV et al. Statins in the prevention and treatment of heart failure: a review of the evidence. Curr Atheroscler Rep 2019; 21:41. https://pubmed.ncbi.nlm.nih.gov/31350612/31350612 10.1007/s11883-019-0800-z PMC6660504

[59] Schonck WAM, Stroes ESG, Hovingh GK et al. Long-term efficacy and tolerability of PCSK9 targeted therapy: a review of the literature. Drugs 2024; 84:165–78. https://link.springer.com/article/10.1007/s40265-024-01995-9 38267805 10.1007/s40265-024-01995-9 PMC10981656

[60] Deswal A, Petersen NJ, Feldman AM et al. Cytokines and cytokine receptors in advanced heart failure: an analysis of the cytokine database from the Vesnarinone trial (VEST). Circulation 2001; 103:2055–9. https://pubmed.ncbi.nlm.nih.gov/11319194/11319194 10.1161/01.cir.103.16.2055

[61] Zoccali C, Mallamaci F. Innate immunity system in patients with cardiovascular and kidney disease. Circ Res 2023; 132:915–32. https://pubmed.ncbi.nlm.nih.gov/37053283/37053283 10.1161/CIRCRESAHA.122.321749

[62] Zoccali C, London G. Con: vascular calcification is a surrogate marker, but not the cause of ongoing vascular disease, and it is not a treatment target in chronic kidney disease. Nephrol Dial Transplant 2015; 30:352–7. https://pubmed.ncbi.nlm.nih.gov/25712936/25712936 10.1093/ndt/gfv021

[63] Boulet J, Sridhar VS, Bouabdallaoui N et al. Inflammation in heart failure: pathophysiology and therapeutic strategies. Inflammation Res 2024; 73:709. https://pmc.ncbi.nlm.nih.gov/articles/PMC11058911/10.1007/s00011-023-01845-6 PMC11058911 38546848

[64] Murphy SP, Kakkar R, McCarthy CP et al. Inflammation in Heart failure: JACC State-of-the-art review. J Am Coll Cardiol 2020; 75:1324–40. 10.1016/j.jacc.2020.01.014 32192660

[65] Macdougall IC, White C, Anker SD et al. Intravenous iron in patients undergoing maintenance hemodialysis. N Engl J Med 2019; 380:447–58. 10.1056/NEJMoa1810742 30365356

[66] Verbrugge FH, Guazzi M, Testani JM et al. Altered hemodynamics and end-organ damage in heart failure: impact on the lung and kidney. Circulation 2020; 142:998–1012. 10.1161/CIRCULATIONAHA.119.045409 32897746 PMC7482031

[67] Pocock SJ, Ariti CA, Collier TJ et al. The win ratio: a new approach to the analysis of composite endpoints in clinical trials based on clinical priorities. Eur Heart J 2012; 33:176–82. https://pubmed.ncbi.nlm.nih.gov/21900289/21900289 10.1093/eurheartj/ehr352

[68] Petrie M, Borlaug B, Buchholtz K et al. HERMES: effects of ziltivekimab versus placebo on morbidity and mortality in patients with heart failure with mildly reduced or preserved ejection fraction and systemic inflammation. J Card Fail 2024; 30:126. 10.1016/j.cardfail.2023.10.024

[69] Chair SY, Chan JYW, Waye MMY et al. Exploration of potential genetic biomarkers for heart failure: a systematic review. Int J Environ Res Pub Health 2021; 18:5904. https://pmc.ncbi.nlm.nih.gov/articles/PMC8198957/34072866 10.3390/ijerph18115904 PMC8198957

[70] Cautela J, Tartiere JM, Cohen-Solal A et al. Management of low blood pressure in ambulatory heart failure with reduced ejection fraction patients. Eur J Heart Fail 2020; 22:1357–65. https://pubmed.ncbi.nlm.nih.gov/32353213/32353213 10.1002/ejhf.1835 PMC7540603

[71] McDonagh TA, Metra M, Adamo M et al. 2021 ESC guidelines for the diagnosis and treatment of acute and chronic heart failure: developed by the Task Force for the diagnosis and treatment of acute and chronic heart failure of the European Society of Cardiology (ESC). With the special contribution of the Heart Failure Association (HFA) of the ESC. Eur J Heart Fail 2022; 24:4–131. https://pubmed.ncbi.nlm.nih.gov/35083827/35083827 10.1002/ejhf.2333

[72] Grobbee DE, Filippatos G, Desai NR et al. Epidemiology and risk factors for hyperkalaemia in heart failure. ESC Heart Fail 2024; 11:1821–40. 10.1002/ehf2.14661 38439165 PMC11287317

[73] De Nicola L, Ferraro PM, Montagnani A et al. Recommendations for the management of hyperkalemia in patients receiving renin–angiotensin–aldosterone system inhibitors. Int Emerg Med 2023; 19:295–306. https://pmc.ncbi.nlm.nih.gov/articles/PMC10954964/10.1007/s11739-023-03427-0 PMC10954964 37775712

[74] Sarnak MJ, Astor BC. Implications of proteinuria: CKD progression and cardiovascular outcomes. Adv Chronic Kidney Dis 2011; 18:258–66. 10.1053/j.ackd.2011.04.002 21782132

[75] Kalay Z, Sahin OE, Copur S et al. SGLT-2 inhibitors in nephrotic-range proteinuria: emerging clinical evidence. Clin Kidney J 2023; 16:52–60. 10.1093/ckj/sfac189 36726436 PMC9871839

[76] van der Aart-van der Beek AB, de Boer RA, Heerspink HJL. Kidney and heart failure outcomes associated with SGLT2 inhibitor use. Nat Rev Nephrol 2022; 18:294–306. https://www.nature.com/articles/s41581-022-00535-6 35145275 10.1038/s41581-022-00535-6

